# Phages infecting *Faecalibacterium prausnitzii* belong to novel viral genera that help to decipher intestinal viromes

**DOI:** 10.1186/s40168-018-0452-1

**Published:** 2018-04-03

**Authors:** Jeffrey K. Cornuault, Marie-Agnès Petit, Mahendra Mariadassou, Leandro Benevides, Elisabeth Moncaut, Philippe Langella, Harry Sokol, Marianne De Paepe

**Affiliations:** 1 Micalis Institute, INRA, AgroParisTech, Université Paris-Saclay, 78350 Jouy-en-Josas, France; 2 MaIAGE, INRA, Université Paris-Saclay, 78350 Jouy-en-Josas, France; 3 Department of General Biology, Federal University of Minas Gerais, Belo Horizonte, Brazil; 4 Sorbonne Universités, UPMC Univ. Paris 06, École normale supérieure, CNRS, INSERM, APHP Laboratoire des Biomolécules (LBM), 27 rue de Chaligny, 75012 Paris, France; 5 Department of Gastroenterology, Saint Antoine Hospital, Assistance Publique – Hopitaux de Paris, UPMC, Paris, France

**Keywords:** Bacteriophages, *Faecalibacterium prausnitzii*, Inflammatory bowel disease, Comparative genomics, Prophages

## Abstract

**Background:**

Viral metagenomic studies have suggested a role for bacteriophages in intestinal dysbiosis associated with several human diseases. However, interpretation of viral metagenomic studies is limited by the lack of knowledge of phages infecting major human gut commensal bacteria, such as *Faecalibacterium prausnitzii*, a bacterial symbiont repeatedly found depleted in inflammatory bowel disease (IBD) patients. In particular, no complete genomes of phages infecting *F. prausnitzii* are present in viral databases.

**Methods:**

We identified 18 prophages in 15 genomes of *F. prausnitzii*, used comparative genomics to define eight phage clades, and annotated the genome of the type phage of each clade. For two of the phages, we studied prophage induction in vitro and in vivo in mice. Finally, we aligned reads from already published viral metagenomic data onto the newly identified phages.

**Results:**

We show that each phage clade represents a novel viral genus and that a surprisingly large fraction of them (10 of the 18 phages) codes for a diversity-generating retroelement, which could contribute to their adaptation to the digestive tract environment. We obtained either experimental or in silico evidence of activity for at least one member of each genus. In addition, four of these phages are either significantly more prevalent or more abundant in stools of IBD patients than in those of healthy controls.

**Conclusion:**

Since IBD patients generally have less *F. prausnitzii* in their microbiota than healthy controls, the higher prevalence or abundance of some of its phages may indicate that they are activated during disease. This in turn suggests that phages could trigger or aggravate *F. prausnitzii* depletion in patients. Our results show that prophage detection in sequenced strains of the microbiota can usefully complement viral metagenomic studies.

**Electronic supplementary material:**

The online version of this article (10.1186/s40168-018-0452-1) contains supplementary material, which is available to authorized users.

## Background

*Faecalibacterium prausnitzii* is one of the most abundant bacterial species of the human gastrointestinal tract (GIT) microbiota. This species is an important producer of butyrate, a short-chain fatty acid with a broad range of beneficial properties on human health [1, 2]. *F. prausnitzii* also exerts anti-inflammatory activity through the production of the MAM protein [3] and provides protection against inflammation in different animal models [3–6]. In humans, a low abundance of this species has been regularly reported in the gut microbiota of inflammatory bowel disease (IBD) patients [6, 7]. Depletion of *F. prausnitzii* in patients has been associated with a higher risk of recurrence of flares [4, 8], possibly in relation with the capacity of this species to modulate T cell responses [6]. Bacteriophages, or phages, the viruses infecting bacteria, might play a role in this depletion [9]. But, although viral metagenomics studies have provided indications that *F. prausnitzii* phages are present in human fecal samples [10], no complete *F. prausnitzii* phage has been characterized to date. Phages can be either virulent or temperate: virulent phages perform only lytic cycles, during which new phage particles are produced and the infected bacteria are killed. Temperate phages can similarly replicate through lytic cycles, but they are also able to enter a latent lysogenic state. During lysogeny, the repressed phage, known as a prophage, is generally integrated into the bacterial chromosome, and the expression of most of its genes is repressed. Nevertheless, a few genes continue to be expressed, including the repressor of the lytic cycle and a class of genes known as morons whose expression often modifies the physiology of the host bacterium. In this way, prophages can modify numerous phenotypes of their bacterial host, such as resistance against phage attack, stress resistance, pathogenicity, or ability to form biofilms [11, 12]. In response to a specific signal, active prophages can switch back to lytic multiplication, a process known as induction. Because of this, active prophages can be highly detrimental to their host in some inducing conditions [13].

Therefore, not only virulent phages but also temperate ones have been suspected to play a role in dysbiosis of intestinal microbiota [14, 15]. To examine this possibility, several viral metagenomic studies of the intestinal microbiota have been performed in recent years [10, 16–18]. However, a major limitation of these studies is the difficulty of viral sequence analysis. Indeed, annotation of viral sequences, and in particular the prediction of the bacterial host, is mainly based on alignment to reference viral sequences in databases [19], and limitations in the viral databases render annotation of most viral sequences difficult if not impossible. Limitations are particularly flagrant concerning phages infecting important groups such as *Ruminococcaceae*, previously known as *Clostridium* cluster IV. There is, to our knowledge, no phage infecting members of this family whose complete genome has been deposited in viral databases. Isolating and describing phages infecting *Clostridiaceae* species such as *F. prausnitzii* is thus a prerequisite for investigation of their potential role in dysbiosis. In this study, we characterize for the first time complete *F. prausnitzii* temperate phages and show that they belong to eight new proposed viral genera. In addition, we show that some of them are either more prevalent or more abundant in the fecal samples of IBD patients compared to healthy controls, suggesting these phages might play a role in the disease.

## Results

### Identification of 23 prophages corresponding to novel proposed genera

Fifteen *F. prausnitzii* genomes, among which 10 were recently published [20], were analyzed with the PHASTER interface (http://phaster.ca), dedicated to prophage detection [21, 22]. Expert inspection retained 23 complete prophages out of the 50 predicted regions, whose borders were manually refined, notably by detecting integrases (Additional file 1). Most integrases belonged to one of the two classical families of phage integrase: tyrosine integrase and large serine recombinase (LSR) (Table 1). The integrase of prophage Lagaffe, however, is atypical since it belongs to the IS30 transposase family, found almost exclusively in transposons. Enzymes of this family were recently described in small temperate single-stranded DNA filamentous phages, and mediate site-specific recombination in a chimeric prophage [23], but had not yet been described in large prophages.

**Table 1:** Main characteristics of the eight proposed *F. prausnitzii* phage genera

Proposed genus	Classification (Virfam)	Bacterial hosts	Prophage name	Strains with complete prophage^a^	Common features				Evidence of activity	
					Genome size, kb	RT	Integrase type	Recombinase	CRISPR spacers^a^	Viral contig
“FPlagaffevirus”	Myoviridae type 1, cluster 6	*F. prausnitzii* *B. hansenii* CM62/1	Lagaffe	A2-165, *B.* *hansenii* 20583, CM62/1	48.3	Yes	IS30 TPase	ERF		Yes
“FPmushuvirus”	Myoviridae type1, cluster 8 “Saltoviridae”	*F. prausnitzii*	Mushu	A2-165, C_4543, C_4574	36.4	Yes	Mu TPase			Yes
		*Ruminococcaceae* bacterium D16	MushuRBD16	R. bacterium D16	36.2	Yes	Mu TPase			
“FPlughvirus”	Siphoviridae type 1, cluster 1	*F. prausnitzii*	Lugh	C_4574, C_4543	34.1		Tyr Int	Sak4	C_4544	
			Lugh4544	C_4544	35.9		Tyr Int	ERF		
			Lugh4542	C_4542	36.4		Tyr Int	ERF		
			LughKLE1255	KLE1255	36.6		Tyr Int	ERF		
			Lugh4540	C_4540	31.1		Tyr Int	Sak4		
			LughL2-6	L2-6	31.2		Tyr Int			
“FPtaranisvirus”	Myoviridae type 1, cluster 6	*F. prausnitzii*	Taranis	C_4574, C_4543	56.0	Yes	LSR	Sak4	C_4541(2), L2/6	
			TaranisL2-6	L2-6	41.9		LSR	Sak4	C_4541	
“FPeponavirus”	Myoviridae type 1, cluster 6	*F. prausnitzii*	Epona	C_4573, C_4575	49.5	Yes	LSR		C_4541, C_4575	
			EponaM21-2	M21/2	48.5	Yes	LSR			
“FPtoutatisvirus”	Myoviridae type 1, cluster 6	*F. prausnitzii*	Toutatis	C_4542	54.7	Yes	LSR		C_4541	
			ToutatisL2-6	L2-6	53.6	Yes	LSR		C_4541	
			ToutatisKLE1255	KLE1255	54.5	Yes	LSR		C_4541, L2/6	Yes
			ToutatisSL3/3	SL3/3	50.1		LSR			
“FPbrigitvirus”	Myoviridae type 1, cluster 7	*F. prausnitzii*	Brigit	C_4644	61.6	Yes	LSR	RecT	C_4541	
“FPoengusvirus”	Siphoviridae type 1, cluster 2	*F. prausnitzii*	Oengus	C_4542	58.4		Tyr Int			Yes

*RT* retron-type reverse transcriptase, *Tpase* transposase, *Tyr int* tyrosine recombinase superfamily, *LSR* large serine recombinase, *CM62/1* unclassified *clostridium* sp. CM62/1

^a^C_ stands for CNCM

**Fig. 1:**
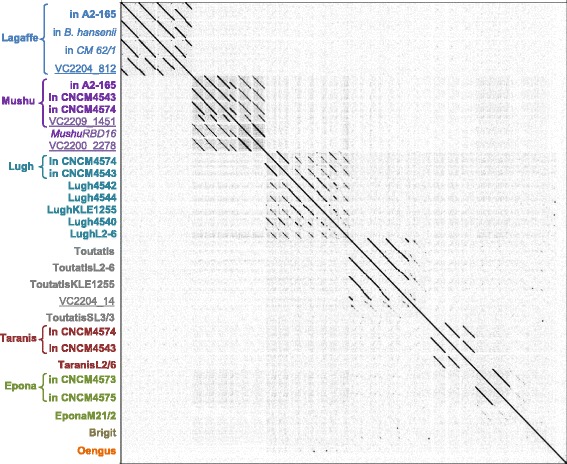
Whole genome dot plot of the 23 *F. prausnitzii* prophages and 7 homologous sequences retrieved from the nr/nt database define six clades and two singletons (Brigit and Oengus, last lanes). Braces group similar prophages, which correspond to the same phage species found in different bacterial genomes. Names beginning “VC” (underlined) correspond to metagenomic viral contigs, and names in italic correspond to prophages in non-*F. prausnitzii* bacterial species

The 23 retained prophages were compared pairwise at the nucleotide level, allowing the definition of six phylogenetic clades (Fig. 1), within which gene synteny was well conserved, and two singletons. The clusters/singletons are well separated from each other, mostly with no gene shared between clusters. None of the predicted prophages had significant nucleotide similarity with a viral genome of the NCBI RefSeq genome database on more than 5% of its genome, indicating they all correspond to new viruses. According to current taxonomic metrics whereby phages within a genus share at least 40% of their proteins, and phages of the same species have more than 95% identity on the whole genome [24, 25], the 23 prophages correspond to 18 species and 8 genera (Additional file 2), for which we propose names (Table 1 and Fig. 1). Of note, Taranis and Toutatis belong to related genera since they share 26% of their proteome, with an overall 39% amino acid identity.

Given the ubiquity of *F. prausnitzii* in human gut microbiota, we investigated whether similar phage sequences had been found in other studies. To this end, we compared the prophage genomes against the NCBI nr database. Prophages Lagaffe, Mushu, and ToutatisSL3/3 have homology to four large contigs assembled from reads obtained by sequencing DNA from virus-like particles (VLPs) isolated from human fecal samples [26] (Fig. 1). In addition, ToutatisL2/6 is homologous to a 9-kb contig assembled from reads of the VLPs of a human fecal sample. This contig had been predicted to correspond to a *F. prausnitzii* prophage [10]. This provides further support for the proposition that these prophage regions are of viral nature. We then looked for CRISPR spacers matching the prophages in the 15 *F. prausnitzii* genomes, and found spacers for eight prophages (Table 1), which is indicative of recent infection of these strains by the respective phages [27]. Overall, indications of activity were obtained for at least one prophage of each proposed genus, as summarized in Table 1.

For six of the eight prophage groups, the host range seemed to be restricted to *F. prausnitzii*, since no similar prophage could be detected by BLASTn in other genomes of the NCBI RefSeq database. On the other hand, prophages with 65% overall sequence identity to Mushu prophage were found in the unclassified *Ruminococcaceae* bacterium D16 (Fig. 1 and Additional file 3) and in the unclassified bacterium SS3/4 (Genbank sequence FP929062.1). Even more striking was the detection of a prophage almost identical to Lagaffe (98% nucleotide identity) in the genome of *Blautia hansenii* DSM 20583 (Fig. 1 and Additional file 3), a bacterium only related to *F. prausnitzii* A2-165 at the order level (they share only 29% of their proteins).

To classify these proposed new phage genera, since they greatly differ from known viruses, we used the Virfam webserver (biodev.cea.fr/virfam). Virfam uses virion protein remote homology and gene synteny to classify phages [28]. All the phages were classified among the Neck type 1, the largest clade of the Caudoviridae order (Additional file 4). Four genera grouped within the cluster 6 of this neck type, which branches deeply in the overall tree and comprises phages that could not be attributed to better-resolved clusters. Phage Mushu belongs to cluster 8, which corresponds to the “Saltoviridae” proposed family, composed of Mu-like transposable phages [29]. Accordingly, Mushu and its relative from the *Ruminococcaceae* bacterium D16 genome have been assigned to this proposed family [29, 30]. Phages from the proposed “FPlughvirus” genus and phage Oengus group in clusters 1 and 2 respectively, with other unclassified Siphoviridae phages infecting Firmicutes. Surprisingly, Brigit phage groups in cluster 7, which typically comprises phages infecting *Enterobacteriaceae*.

### Functional annotation of prophage genes reveals a high prevalence of diversity-generating retroelements

**Fig. 2:**
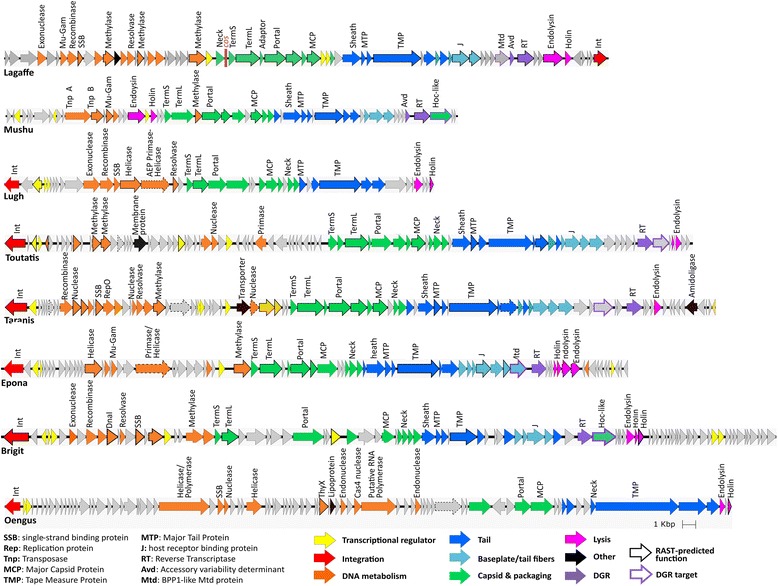
Genomes of each type phage. Genes whose product were predicted by RAST rather than by Phagonaute are indicated by a bold outline, and genes whose RAST-predicted function was changed in this study are indicated by a dashed outline

Within each proposed genus, we chose one representative prophage, which we subsequently refer to as the “type phage” for the group. Each type phage was annotated by using the Phagonaute webserver (genome.jouy.inra.fr/phagonaute), which uses remote homology searches and gene synteny information [28, 31]. We improved the functional predictions as compared to the previous automatic annotation using RAST server by 2.5- to 5-fold (Fig. 2). All phage functional modules were detected in each prophage, providing further indication that these prophages correspond to complete phage genomes.

A remarkable feature of these genomes is the high prevalence of diversity-generating retroelements (DGRs). DGRs generate variability in target genes through a reverse transcriptase (RT)-mediated mechanism that introduces nucleotide substitutions at defined locations in specific genes (reviewed in [32]). In this process, adenine residues are converted to random nucleotides in a unidirectional process from a donor template repeat (TR) to a recipient variable repeat (VR) present in the target gene. TR and VR sequences were identified in each prophage type coding for an RT (Additional file 5). As with other DGRs, TR/VR sequences were between 100 and 135 bp long, they differed almost exclusively at sites corresponding to TR adenines, and the VRs were located at the 3′ end of protein-coding genes. We then searched for analogues of Avd, a low-molecular-weight protein required for mutagenic homing in the prototype DGR of the *Bordetella pertussis* phage BPP1 [33]. Homologs of Avd were detected in Mushu and Lagaffe genomes only (Fig. 2). In other phages, DGRs might function with another small protein, as has previously been suggested for other DGRs [34]. In Lagaffe and Epona, the putative target gene encodes a protein belonging to the formylglycine-generating enzyme (FGE) superfamily, which includes the BPP1 tail protein MTD, implicated in bacterial recognition [35]. Mushu and Brigit DGR target genes code for a Hoc-like capsid decoration protein, which in T4 improves the binding to intestinal mucus [36]. In Toutatis and Taranis, no putative function could be attributed to the DGR target.

### Lagaffe and Mushu produce virions

**Fig. 3:**
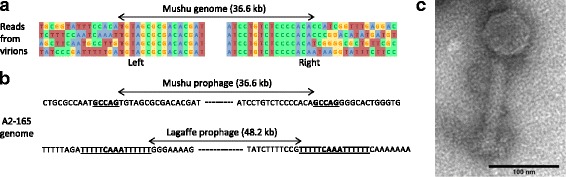
Mushu and Lagaffe are active phages. **a** Fragments of eight arbitrarily chosen reads of the two extremities of Mushu encapsidated DNA, showing the variable bacterial sequences on each side, typical of transpositional replication. **b** Direct repeats (underlined) on both sides of the prophages are present in the A2-165 genome. **c** Photograph of Mushu virion obtained by transmission electronic microcopy

We next evaluated the activity of Lagaffe and Mushu in laboratory conditions by isolating virions from the supernatant of a culture of strain A2-165 and sequencing the encapsidated DNA. We obtained 19,702 and 30,274 reads mapping to the Lagaffe and Mushu prophages, which correspond respectively to an 8- and 16-fold higher coverage than the rest of A2-165 genome (Additional file 6). This demonstrated that the two prophages produce virions and that there is no other active prophage in the A2-165 strain. Analysis of Mushu reads confirmed that it replicates by transposition: at the extremity of the Mushu genome, reads were flanked by variable host DNA sequences on both sides (Fig. 3a). This particular configuration is similar to that of *E. coli* Mu phage encapsidated DNA, and results from packaging by a full head mechanism of integrated viral copies spread randomly through the host genome [37]. Analysis of reads also revealed the Mushu and Lagaffe precise integration sites in the A2-165 chromosome (Fig. 3b). Mushu integration site presents the typical 5 bp direct repeat of some Mu-like phages [37]. More surprisingly, Lagaffe is also flanked by direct repeats, whereas a sequence composed of inverted repeats is expected to form the integration site of IS30 transposases [23]. Analysis of Lagaffe reads with the PhageTerm tool [38], dedicated to the detection of the strategy used by phages to package their genome into capsids, identified the *cos* region of Lagaffe, upstream of the terminase gene *termS*.

For both phages, reads spanning the VR region of the DGR are more variable than the rest of the genome, an evidence of DGR activity (Additional file 5C). The level of variability observed in Mushu is however remarkable, since the phage genomes sequenced result in theory from a single cycle of multiplication (they are produced following induction in lysogenic cells), and suggests a very high efficacy of TR-to-VR transfer in this DGR.

Lagaffe virions could also be detected in *Blautia hansenii* DSM 20583 cultures by quantitative PCR (Table 2), which demonstrates the remarkable polyvalent nature of this phage. Indeed, usually, phages are restricted to strains of the same species or sometimes the same genus [39]. Only few cases have been reported of a phage infecting bacteria from different families, similar to Lagaffe [40, 41]. Finally, observation by electron microscopy of A2-165 culture supernatant revealed Mushu virions, with a head diameter of 61 ± 1 nm and a tail of length of 134 ± 2 nm and width of 22 ± 1 nm (Fig. 3c). Due to its very low concentration in A2-165 culture (3 × 10^5^ ml^−1^, Table 2), intact Lagaffe virions could not be observed.

**Table 2:** Lagaffe and Mushu virion concentration in culture supernatants and in mouse feces

	Virions/ml or virions/g with *F. prausnitzii* A2165			Virions/ml in *B. hansenii* cultures
	Cultures	Healthy mouse gut	Inflamed mouse gut	
Lagaffe	3 ± 0.3 × 10^5^	< 1 × 10^6^	< 1 × 10^6^	6 ± 3 × 10^5^
Mushu	8 ± 2 × 10^6^	9 ± 2 × 10^6^	7 ± 2 × 10^6^	

Virions were quantified by qPCR on encapsidated genomes. Intestinal inflammation in mouse was induced by adding DSS to drinking water. Cultures correspond to saturated 24 h cultures grown in LY-BHI

### Lagaffe and Mushu prophages are not inducible by DNA damage

To increase virion titers and search for inducing signals, A2-165 *F. prausnitzii* cultures were treated with different concentrations of three potent inducers of phage lytic cycle: mitomycin C, hydrogen peroxide, and bile salts (cholate/deoxycholate [1:1]) [13, 42]. Mitomycin C in particular causes DNA damage, which is a general signal for prophage derepression. However, none of the treatments significantly increased virion concentrations (Additional file 7). For each compound tested, the highest dose tested almost abolished bacterial growth, indicating that they are toxic for *F. prausnitzii* and that the absence of prophage induction is not due to too low concentrations of potent inducers. Lagaffe and Mushu are thus probably not inducible by DNA damage, similar to most Mu-like phages.

### Mushu virions are produced in the mouse gastro-intestinal tract but are not increased by colitis

We next investigated the production of A2-165 prophages in the mouse gastro-intestinal tract (GIT). Indeed, recent studies have shown increased prophage induction in the murine GIT, as compared to in vitro conditions [43, 44]. In order to obtain a colonization level of *F. prausnitzii* sufficient to detect virions in feces, we used dixenic mice colonized with *F. prausnitzii* A2-165 and *Escherichia coli*. In these mouse feces, the Lagaffe virion concentration was too low to be detected, but Mushu virions were present in similar concentrations to those observed in vitro (Table 2 and Additional file 8). Of note, however, the potential binding of virions to bacterial cells and intestinal mucus prevents the direct deduction of prophage induction rates from the concentrations of free virions. We then monitored the effect of moderate inflammation on the prophage induction rate. To mimic moderate inflammatory conditions, dextran sodium sulfate (DSS) was added at 2% to drinking water for 7 days. This treatment resulted in mild inflammation but did not increase virion concentrations in mouse feces (Additional file 8).

### Some *F. prausnitzii* phages are more abundant in the gut viromes of IBD subjects

**Fig. 4:**
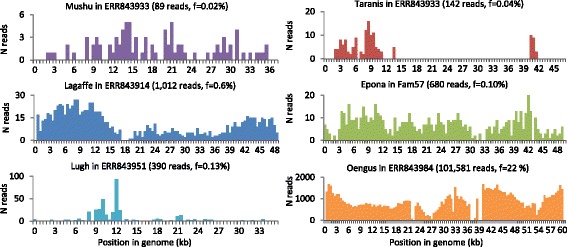
*F. prausnitzii* phages are present in intestinal viromes. Read coverage for six type phages in samples with the best abundance and coverage from the datasets of Norman et al. [18] and Reyes et al. [52]. Number of aligned reads (ordinate) is represented as a function of position in the phage genome (abscissa). Phage name, virome name, total number of aligned reads, and ratio of aligned to total reads are shown for each panel

To evaluate the activity of the identified prophages in the human GIT, we investigated the sequences obtained from the viral fraction of 171 fecal samples from a large cohort of 52 IBD patients and 21 healthy controls [18]. Alignment by BLASTn of reads from this dataset to 17 of the identified prophages retrieved a large number of hits (ToutatisL2/6 being very close to Toutatis, 96% identical over 80% of its length; it was not included in this analysis). Altogether, virome reads mapping to at least one of the eight type phages represented 0.02% of all reads in healthy samples and 0.30% in IBD samples (mean of the proportion of reads matching on *F. prausnitzii* type phages over total number of reads). Remarkably, reads mapping to phage Oengus represented 22% of reads in one sample (Fig. 4, sample ERR843984). In that sample, we could assemble the matching reads into a contig covering 93% of Oengus genome with 99% identity. Yet matching read coverage was not always even on the prophages (Fig. 4), suggesting the presence of phages only sharing genome fragments with the phages of this study. Concerning phages of the “FPlughvirus” proposed genus, this may be due to the high level of genomic mosaicism in this genus, i.e., the alternation of regions of high identity with regions of low identity between phage genomes (Additional file 9). Of note, various genes coding for homologous recombination proteins, involved in phage mosaicism, were found in this genus (Table 1) [45, 46].

**Fig. 5:**
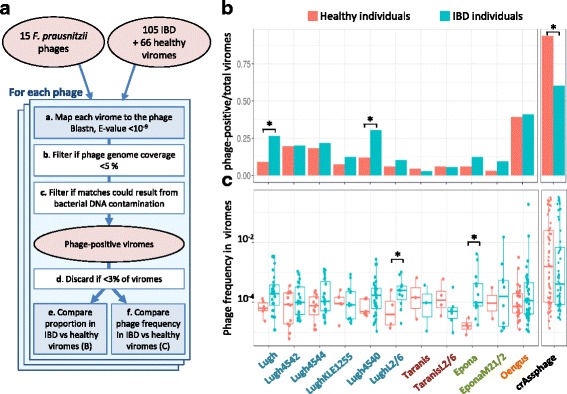
*F. prausnitzii* phages in healthy versus IBD-diseased individuals from the dataset of Norman et al. [18]. **a** Workflow of the procedure. a To infer the presence of a phage in a given sample, virome reads were aligned to the phage genome, and hits showing greater than 75% identity were retained. b If hits of a given sample were concentrated on less than 5% of the phage genome, the sample was not considered further for this phage. c The number of hits was compared to the maximal theoretical number of hits that could result from the measured bacterial DNA contamination. If the number of hits was significantly higher, the sample was classified as phage-positive. d If phage-positive samples represent less than 3% of total samples, this phage was not further studied. e The proportion of phage-positive viromes is compared to IBD versus healthy viromes. f In phage-positive viromes, phage frequency is compared to IBD versus healthy samples. **b** Fraction of phage-positive samples according to the health status of individuals. Three phages are significantly more prevalent in IBD-diseased than in healthy individuals. The highly prevalent crAssphage was included as a positive control in the analysis. **c** Phage abundance (read frequency) in positive samples according to the health status of individuals. Two phages, Epona and LughL2/6, are significantly more abundant in IBD patients than in healthy individuals

We then wished to evaluate whether *F. prausnitzii* phages are significantly more prevalent and/or abundant in fecal samples of healthy individuals as compared to those of IBD patients. To this end, for each phage, we first determined phage-positive viral samples, i.e., the samples that unambiguously contain that phage or related ones. A flowchart of the procedure is represented in Fig. 5a. Since Lagaffe and Mushu relatives infect species other than *F. prausnitzii*, whose link with IBD is not known, they were removed from the analysis. Eleven of the 15 investigated phages were present in more than 3% of samples, and one of them, Oengus, was found in up to 40% of samples (Fig. 5b). Two “FPlughvirus” phages were statistically more prevalent in IBD samples than in healthy controls (Fig. 5, two tailed two-proportion *z* test, *p* < 0.05). We then compared the proportion of reads homologous to each phage in phage-positive samples by fitting a logistic regression with status (healthy/IBD) as fixed effect and patient as mixed effect. Overall, all phages except those from the “FPtaranisvirus” proposed genus are more abundant in the IBD samples than in the healthy ones, although the difference reaches significance only for LughL2/6 and Epona (Fig. 5c, *p* value < 0.05). Since *F. prausnitzii* populations are generally less abundant in IBD patients than in healthy controls [7], the higher occurrence and proportion of some *F. prausnitzii* temperate phages suggest that they are more active in the microbiota of IBD patients and that this activity is related to the depletion of *F. prausnitzii*.

## Discussion

*F. prausnitzii* genomes are particularly rich in prophages: in this study, we found that 85% of them harbor at least one complete prophage, as opposed to only 46% of all completely sequenced bacterial genomes [47]. The 23 identified complete prophages in this study correspond to 18 different phages, which can be grouped into eight new viral genera. Thanks to experimental and in silico investigations, we found evidence of activity for at least one member of each proposed genus. Their novelty and diversity are striking and greatly enrich the viral databases, which are at present highly skewed towards phages infecting *proteobacteria* or other pathogens*,* which together account for more than 75% of phages in the NCBI viral genome database (as of December 2017). The novelty of the viral proteins coded by *F. prausnitzii* phages should not only improve the analysis of viral metagenomic sequences but might also lead to biotechnology applications, such as genetic engineering of *F. prausnitzii*.

A very high proportion *F. prausnitzii* phages possesses DGRs: these diversification systems are present in 10 out of the 18 phages. By comparison, only 0.05% of the 876 phages present in the Phagonaute database (comprising all fully sequenced phage genomes available at the NCBI as of May 2014) possess DGRs. This high prevalence in *F. prausnitzii* phages might explain the previously observed high frequency of DGRs in intestinal viromes [26]. By generating variants of target genes, DGRs presumably confer selective advantages to the phages, such as the ability to infect bacteria with different variants of the surface component used as receptor by the phage, as has been shown in the case of *B. pertussis* phage BPP1. In this phage, the DGR-mediated diversification of the MTD protein generates at low-frequency phage variants able to infect bacteria with receptors different from those of the original host [33]. In phages Lagaffe and Epona, the proteins diversified by the DGR are homologous to BPP1 MTD, so their diversification probably also facilitates phage adaptation to different bacterial receptors. This property would provide a selective advantage to the phage either in the case of a preexisting high bacterial surface receptor diversity or in conditions of continually evolving bacteria in the context of an ongoing arms race between phage and their hosts. In phages Brigit and Mushu, by targeting Hoc-like capsid decoration proteins, the DGR system could improve the binding of virions to mucus, a strategy that has been suggested to increase the probability for phages to encounter their bacterial host [36]. Whatever the role of these DGRs, they contribute to the generation of microdiversity regions in the genomes of intestinal phages, which notoriously complicates the assembly of reads, and hence the analysis of GIT viral metagenomes [48].

We found that the *F. prausnitzii* temperate phages described here are present in 5 to 40% of the human fecal samples from the study of Norman and colleagues [18]. Despite their relatively low abundance in most samples of this dataset, they can represent up to 22% of total viral reads (Additional file 10). Most importantly, some of them are either more prevalent or more abundant in samples from IBD patients compared to that of healthy controls. This result is striking since IBD patients have generally a lower abundance of *F. prausnitzii* in their microbiota [6, 7]. This suggests that this higher prevalence or abundance is not just the consequence of a higher abundance of their bacterial host but rather that these *F. prausnitzii* prophages are more active in the microbiota of IBD patients as compared to healthy controls. This phage activity could aggravate or even trigger *F. prausnitzii* depletion. Since this depletion is highly suspected to participate in intestinal inflammation, our results suggest for the first time a role of *F. prausnitzii* phages in IBD.

## Conclusion

Here, we describe for the first time complete *F. prausnitzii* temperate phage genomes. These phages belong to the new viral genera, and most of their proteins have only remote homology with previously described viral proteins. The novelty of *F. prausnitzii* phages warrants great enrichment of databases, which should in turn significantly improve annotation of viral gut metagenomic sequences. In addition, we show that some of these *F. prausnitzii* phages are either more prevalent or more abundant in the fecal samples of IBD patients compared to that of healthy controls, suggesting that these phages might play a role in the disease. Future mechanistic exploration of the nature of phage-bacteria interactions is now necessary to elucidate the role of temperate phages in dysbiosis.

## Methods

### Prophage detection and comparison

*F. prausnitzii* genomes listed in Additional file 1 were analyzed with PHASTER (http://phaster.ca). “Intact” and “questionable” regions were then validated by manual inspection. We retained regions with genes in each of the following modules: replication, capsid, lysis, and lysogeny, and no genes specific of integrative plasmids or insertion sequences. Of note, several prophage regions were on borders of contigs, suggesting that some complete prophages could have been missed because of splitting between two contigs and that some of the retained prophages might lack some genes. Prophage borders were refined by searching for phage integrase and phage-specific genes by BLASTp against the nr NCBI database. In several cases, the ORF corresponding to the integrase had not been detected automatically and was added manually. For type prophages, few other ORFs were also added. In addition, repeats on both sides (attL and attR sites) were searched for. They could be identified only in prophages encoding a tyrosine integrase, which is not surprising since tyrosine integrases generally use longer identical repeat than serine integrases. A multi-fasta file containing all prophage nucleotide sequences is available in the Additional file 11. The type phage genomes were deposited in the NCBI viral database (accession numbers in Additional file 1) and are consultable on the Phagonaute webserver (genome.jouy.inra.fr/phagonaute).

Genomic regions homologous to the 18 prophages were searched for in the nr/nt nucleotide collection of the NCBI by BLASTn, with the discontinuous megaBLAST default parameters. Sequences producing alignments covering more than 5% of the query were retained. Whole genome dotplots were realized with Gepard [49], using a length of 11 nt for minimal exact matches. CRISPR spacers were searched for with CRISPRDetect [50], using default parameters.

### Shared proteins between prophage pairs

For each prophage pair to be compared, all proteins of the first phage were compared to the proteins of the second one (BLASTp, *E* value < 10^−3^), and best hits were considered shared proteins. The proportion of shared proteins was then calculated by dividing the number of shared proteins by the total protein number (minimal value of the pair), and the average identity percent of the shared proteins was computed. To select an outgroup phage for each proposed genus, all proteins of the type phage were compared to the viral protein database subset of the NCBI. The most frequent subject phage among the top 10 results for each protein was selected as the outgroup phage.

### Gene product prediction

Gene function prediction for type prophages was achieved by combining Virfam [28] and Phagonaute [31] web tools. Concerning Virfam, default homology thresholds were used for recombinase and connector proteins. In Phagonaute, the proteins having a HHsearch hit against Pfam (*P* > 95%) were annotated with the Pfam information. Otherwise, the protein was searched against all other phage proteins (probability threshold of 95% and two iterations of HHsearch) and annotated using the most prevalent annotation. If no homolog with experimentally validated function was retrieved by the second iteration, the word “putative” was indicated in the Genbank file.

### Virome read treatments

Reads from 171 viromes extracted from feces of healthy subjects and patients with inflammatory bowel diseases [18] were downloaded from the EBI (fastq files, accession numbers ESR698653-ESR699023). Paired-end reads were treated with trimmomatic [51] to remove low-quality reads and unclip primer sequences present at the 3′ ends of reads, using the following command ILLUMINACLIP:TruSeq3-PE.fa:2:30:10 SLIDINGWINDOW:4:15 MINLEN:36. Reads were then dereplicated with fastx uniques from the usearch9 suite. Since ~ 30% of reads were no longer paired after the trimmomatic treatment, only one read from each pair was used for mapping analyses, to remain quantitative and avoid over-representation of some phage sequences compared to others. Average read length was 250 bp. To complement this analysis, we also analyzed the viral contigs generated in the Reyes et al. study [52]. In this study, fecal viruses from 20 Malawian families were extracted at different time points and sequenced by 454 shotgun pyrosequencing. A cross-assembly strategy yielded 17,696 contigs, among which 780 had a size above 10 kb. These contigs were downloaded from ENA (PRJEB 9818) and compared with the prophages by megaBLAST alignments. Four of these contigs (> 10 kb) shared 93–97% identity to Epona, and together, the similarity regions covered 73% of Epona, but each of these contigs also contained large segments dissimilar to Epona. All four contigs originated from reads of family 57. These reads were therefore downloaded from ENA (PRJEB 9818, samples ERS795750 to 66), quality treated with trimmomatic, and dereplicated with fastx_uniques. This generated 694,650 reads, among which 680 mapped on Epona, whereas only 71 mapped to bacterial *F. prausnitzii* DNA (see the mapping method below and Fig. 4).

### Quantification of *F. prausnitzii* phages in viromes

The proportion of reads homologous to each prophage was determined with BLASTn (*E* value < 10^−9^, > 75% identity). To account for different genome lengths, read counts were normalized by dividing them by genome length and then multiplying the result by the average genome length (50 kb). This allows comparing phage read proportions as if all phages had a genome of length 50 kb. Since temperate phages can also be present in the bacterial DNA as prophages, we verified the level of bacterial DNA contamination of samples. An analysis of reads mapping to 16S rRNA genes with Bowtie2 [46] indicated that the vast majority of the 171 viromes had a low proportion of bacterial DNA: 166 out of 171 had less than 10^−4^ reads matching to 16S rRNA sequences. Two additional specific controls of *F. prausnitzii* DNA presence were included for the mapping analysis: a 50-kb segment corresponding to the beginning of the *F. prausnitzii* A2-165 Genbank file and a 50-kb segment encoding mainly metabolic genes (coordinates 1,451,689–1,501,688). Samples were classified as phage-positive for a given phage when the proportion of reads homologous to that phage was significantly higher than the proportion of reads homologous to these two bacterial regions (one tailed two-proportion *z* test, *p* < 0.05). As a control of the method, we included the highly prevalent crAssphage in the analysis and found that it is more prevalent in healthy controls than in IBD patients (Fig. 5a, two-tailed two-proportion *z* test, *p* < 0.01), in agreement with previously published results [17]. Inspection of prophage coverage within each virome (number of reads per 500 bp interval) revealed three situations: (i) the prophage was largely covered (> 50% length, see Fig. 4), (ii) coverage was partial (Taranis and Lugh in Fig. 4); and (iii) matches were concentrated into 1–2 contiguous prophage intervals. We excluded these last cases (less than three non-overlapping windows of 500 bp, corresponding to at most 5% of the genome) that correspond to spurious matches from various phages, mapping to a conserved region, such as an anti-repressor or an integrase gene. Additionally, to minimize the impact of these spurious matches, the median and average values of matches among all 500 bp intervals with a non-null amount of matches were computed for each phage/sample pair, and in cases were average was 10-fold higher to median, intervals displaying a number of matches above 10 times the median were considered spurious and replaced by an arbitrary value of 10 times the median. The final abundance matrix and coverage value for each phage/virome pair is reported in Additional file 10.

### Viral reads assembly for phage Oengus

Given the elevated abundance of Oengus in virome ERR843984, all reads from this virome mapping on Oengus were retrieved (*n* = 193,414) and assembled with SPAdes (version 3.9) [53], using the following options: -k 21,33,55,77,99,127 --careful --only-assembler. This generated a 53.7-kb contig 99% identical to Oengus, covering 93% of the prophage, with a mean coverage of 322.

### Sequencing of the DNA present in virions produced by the A2-165 strain

Five hundred milliliters of A2-165 culture grown overnight in LY-BHI (brain-heart infusion medium supplemented with 0.5% yeast extract [Difco], cellobiose (1 mg/ml [Sigma]), maltose (1 mg/ml [Sigma]), and cysteine (0.5 mg/ml [Sigma])), in an anaerobic chamber filled with 90% N2, 5% CO2, and 5% H2, was centrifuged at 5,200*g* for 15 min at 4 °C. Supernatant was recovered and centrifuged at 5,200*g* for 30 min at 4 °C. This step was repeated with 1 h of centrifugation. Polyethylene glycol (PEG 8000) and NaCl (Sigma) were then added to the resulting supernatant to a final concentration of 10% and 1 M respectively. The resulting mixture was kept at 4 °C overnight and then centrifuged at 5200*g* for 1 h at 4 °C. Supernatant was removed, and the phage pellet was resuspended in 2 ml of SM buffer (100 mM NaCl, 8 mM MgSO_4_, 50 mM Tris pH 7.5). Sample was then treated with 0.25 μg of RNAse A (Sigma) and Dnase I (Sigma) at 37 °C for 1 h. Phage DNA was extracted using Promega kit Wizard® DNA Clean-up system. Sequencing was performed with the Ion proton sequencing technology. Alignment of obtained reads was performed using bowtie2 (-N 1 –L 32) and then visualized with Tablet, using default parameters [54]. We obtained 204,837 reads mapping to the A2-165 genome, of which 19,702 and 30,274 reads aligned to the Lagaffe and Mushu prophages respectively.

### Prophage induction assay and quantitative PCR

Quantification was made as previously described [44]. Briefly, phage particles from mouse feces (see below) or from 10 ml of overnight culture in LY-BHI were recovered by PEG precipitation, as described above. For induction assays, overnight cultures of *F. prausnitzii* A2-165 were diluted in LY-BHI 25-fold. When culture absorbance reached 0.2, potent inducers were added at final concentrations of 1 or 5 μg/ml for mitomycin C (Sigma, Ref: M4287), 0.1 and 0.05% for bile salts (Sigma 48305), and 100 μM and 10 μm for hydrogen peroxide (Sigma H1009). Five hours after induction, phages were recovered from culture supernatants by PEG precipitation as described above. Samples were then treated with 10 U of Turbo DNase (Ambion) for 1 h at 37 °C and incubated at 95 °C for 30 min in 0.2 ml PCR tubes. qPCR on 100-fold diluted samples in water was performed using the Takyon ROX SYBR Mastermix blue dTTP kit (Eurogentec) and the StepOnePlus real-time PCR system (Applied Biosystem). Phage and bacterial concentrations were determined using specific primer pairs (Additional file 12). Copy numbers were calculated with a standard curve built with bacterial DNA containing the prophages, whose concentration was estimated with Qubit (Thermo Fisher). The reaction mix was the following: 7.5 μl Takyon mix, 0.9 μl dH2O, 0.3 μl of each primer (200 nM final concentration), and 6 μl of DNA diluted in H_2_O. The PCR conditions were the following: 95 °C 15 s, 58 °C 45 s, and 72 °C 30s for 45 cycles, 72 °C 5 min, followed by melting curves. Results were analyzed using the StepOne Software 2.3.

### Mouse colonization experiments

Germfree 7- to 8-week-old C3H/HeN mice (female) were obtained from the germfree rodent breeding facility of Anaxem-Micalis (INRA, Jouy-en-Josas, France). Animals were kept in flexible-film isolators (Getinge-La Calhène) in standard cages (two to five mice/cage) with sterile wood shavings as animal bedding. Mice were given free access to autoclaved tap water and to standard diet R03-40 (Scientific Animal food and Engineering, Augy, France), sterilized by gamma irradiation at 45 kGy (IBA Mediris). Isolators were maintained under controlled conditions of light (12 h), temperature (20 to 23 °C), and humidity (45 to 55%). To obtain dixenic *E. coli*/*F. prausnitzii*-diassociated mice, germfree mice were orally inoculated with a fresh culture of *E. coli* LF82 (10^8^ to 10^9^ CFU/ml). Forty-eight and 72 h later, 10^9^ CFU of *F. prausnitzii* A2-165 in 0.2 ml were administered by intragastric gavage to mice pretreated with sodium bicarbonate (0.2 M, 0.1 ml by intragastric gavage, given 10 min before inoculation of bacteria). To induce colitis, 21 days after *F. prausnitzii* colonization, mice were given drinking water supplemented with 2% (*w*/*v*) dextran sulfate sodium (DSS; MP Biomedicals) for 7 days. Body weight, blood in stool, and stool consistency were monitored daily. Phage particles were recovered from one or two frozen fecal pellets unfrozen on ice for 10 min and diluted 40-fold in cold PBS. Re-suspended feces were kept on ice for 5 min with regular agitation prior to centrifugation for 10 min at 5251*g* at 4 °C. Supernatants were recovered and filtered through a 0.2-μm filter (PALL Corporation Acrodisc PF syringe filter). Samples were supplemented with 10% PEG 8000 (Sigma) and 0.5 M NaCl (Sigma). After incubation overnight at 4 °C, phage particles were harvested by centrifugation (5250*g* for 1 h at 4 °C with a swinging rotor). Supernatant was carefully removed, and pellets were re-suspended in 100 μl of SM buffer (100 mM NaCl, 8 mM MgSO4, 50 mM Tris pH 7.5) for 1 h at 4 °C.

### Transmission electronic microscopy

Phages were concentrated by ultracentrifugation (20,000*g*, 1 h, 4 °C), starting from 500 ml of *F. prausnitzii* A2-165 culture. The phage pellet was resuspended in 100 μl of MgSO_4_ 10 mM. Droplets of this preparation were directly placed on Formvar carbon-coated grids for 5 min. The grids were stained with 1% uranyl acetate and then viewed for TEM using a HITACHI HT 7700 (Elexience, France) at 80 kV. Microphotographies were acquired with a charge-coupled device camera AMT.

## Additional files

Additional file 1: Accession numbers of the bacterial and type phage genomes and bacterial coordinates of all prophages. (DOCX 22 kb)

Additional file 2: Similarity matrices of phage proteins within each proposed genus. For each pair of phages, the percentage of shared proteins as well as their mean amino acid identity is indicated. For each proposed genus, the closest known phage was included in the analysis, and its host is indicated. Numbers in brackets indicate the number of ORF per genome, and most of the variability within a group is due to annotation errors. Numbers in bold indicate highly homologous prophages, which were considered to correspond to the same species. (DOCX 28 kb)

Additional file 3: Alignment of Mushu and Lagaffe with close relatives. A) Lagaffe prophages. Remarkably, Lagaffe prophage is present in the genome of *B. hansenii* DSM20583, only distantly related to *F. prausnitzii*. Alignment with a viral contig obtained from metagenomic reads suggests that the packaging site is just before the terminase gene. B) Mushu prophage is present in three *F. prausnitzii* isolates. Its synteny is perfectly conserved with a distantly related prophage in the genome of the *Ruminococcaceae* bacterium D16 (RBD16). (PPTX 466 kb)

Additional file 4: Classification of *F. prausnitzii* phage genera by Virfam. Classification of *F. prausnitzii* phage genera by Virfam (*biodev.cea.fr**/virfam/*)*.* Four genera fall into the less resolved cluster, the cluster 6. (PPTX 913 kb)

Additional file 5: DGRs in prophages. A) The position of TR and VR regions in phage genomes and the percentage of nucleotide identity between the sequences (% id), as well as the repeat length, are indicated for each type phage. In all cases, the differences are restricted to adenine residues, and the target genes containing the VR are typical of DGR. B) Genetic organization of DGRs of phages Lagaffe and Mushu, and alignment of their template repeat (TR) and variable region (VR). Red bases highlight point mutation between the two regions. Positions are given relative to the beginning of the prophage, except for Lagaffe (beginning of the encapsidated form of the genome). As in other VR/TR repeats, mismatches concern only adenine residues. C) Mapping of sequence reads of encapsidated phage DNA reveals the high variability in the VR region of both phages. Illustrations were obtained using Tablet [54]. Note that in the case of Lagaffe, the sequence corresponds to the complement of the VR sequence in B). (PPTX 194 kb)

Additional file 6: Read coverage of Lagaffe and Mushu prophages. Coverage of the bacterial DNA is represented by surrounding regions of the two prophages. Numbers correspond to positions in the A2-165 genome. Coverage pictures were obtained using Tablet [54]. (PPTX 141 kb)

Additional file 7: Lagaffe and Mushu virion concentrations in culture supernatants. Quantification of Mushu and Lagaffe virions in different conditions. Numbers represent means of genomes/ml ± s.e.m. of two to five independent experiments. Absorbance of the culture (O.D_600nm_) reflects the toxicity of the compound on bacterial growth. (DOCX 13 kb)

Additional file 8: Lagaffe and Mushu virion concentration in the feces of mice. A) *F. prausnitzii* concentration (dark blue and dark red) and Mushu virion concentration (light blue and light red) in the feces of mice, as determined by quantitative PCR. DSS treatment does not modify phage and bacteria population levels compared to untreated mice. B) Variation of mice weight with time. Dots represent the average of five mice; bars represent S.E.M. Mice given DSS in drinking water loosed weight, a sign of inflammation. C) Concentration of lipocalin, a marker of intestinal inflammation [55], in mice feces. Concentration was estimated on feces after 7 days of DSS ingestion, using R&D ELISA kit (DY1857) and following manufacturer protocol. The DNA of fecal bacteria was extracted from feces resuspended in 500 μl of PBS. Suspension was centrifuged 1 min at 500*g*. Supernatant containing bacteria was recovered and mixed with 250 μl of lysis buffer (200 mM NaCl, 20 mM EDTA, 5% SDS), 250 μl of phenol/choloform/isoamyl alcohol (25:24:1;[pH 8.0], Sigma-Aldrich) and half of a tube of silica beads (100 μM MP Bio, Lysing matrix B). Bacteria were lysed using Fast-prep MPBio (5.5, 4 times 30 s). Samples were then centrifuged (13,000*g*, 3 min, 20 °C) and the aqueous phase was recovered. Four hundred microliters of chloroform/isoamyl alcohol(24:1) was added, mixed vigorously and centrifuged (13,000*g*, 3 min, 20 °C). DNA was precipitated with two volumes of ethanol and sodium acetate at 0.3 M final. (PPTX 42 kb)

Additional file 9: Whole genome dotplot of “FPlughvirus” phages. Whole genome dotplot of the six genomes of the proposed “FPlughvirus” genus reveals mosaicism. Phages share regions of high identity interspaced by regions without homology. The dotplot was realized with Gepard. (PPTX 123 kb)

Additional file 10: Number of virome reads homologous to each prophage for all 171 viromes. Number of reads homologous to each prophage (N reads), as determined with BLASTn (*E* value < 10^−9^, > 75% identity). For each phage/virome pair, the number of 500 bp interval covered by reads (N int) is also reported. (XLSX 81 kb)

Additional file 11: Genome sequences of the 18 *F. prausnitzii* prophages (multifasta format). multifasta file of the 18 *F. prausnitzii* prophages. (FA 976 kb)

Additional file 12: Sequences of oligonucleotides used for quantitative PCR. (DOCX 15 kb)
