# Supplementary material for: Phages infecting Faecalibacterium prausnitzii belong to novel viral genera that help to decipher intestinal viromes
Source: Microbiome. 2018 Apr 3;6:65. doi: 10.1186/s40168-018-0452-1 (PMC5883640; doi:10.1186/s40168-018-0452-1)
Supplement: Supplementary file 1 — Accession numbers of the bacterial and type phage genomes and bacterial coordinates of all prophages. (DOCX 22 kb) [file 40168_2018_452_MOESM1_ESM.docx]

| **Bacterial genomes** | | | | |
| --- | --- | --- | --- | --- |
| Phylogroup | **strain** | **Contig accession number** | **Prophage** | |
|  |  |  | Name | Predicted position* |
| *F. prausnitzii*  Phylogroup I | CNCM-4644 | NMTZ01000027 | Brigit | 234,677-296,392 |
|  | CNCM-4546 | NMTV00000000 | - |  |
|  | M21/2 | ABED02000025 | EponaM21/2 | 172,577-221,041 |
|  | CNCM-4573 | NMTW01000053 | Epona | compl(56,066-105,638) |
|  | SL3/3 | FP929046 | ToutatisSL3/3 | 2,692,869-2,742,990 |
| *F. prausnitzii*  Phylogroup II B | A2-165 | CP022479 | Lagaffe | 787,801-836,061* |
|  |  |  | Mushu | 2,972,083-3,008,557* |
|  | CNCM-4543 | NMTT01000002 | Mushu4543 | compl(306,388-342,983) |
|  |  | NMTT01000003 | Lugh | 183,976-218,050 |
|  |  |  | Taranis | 381,591-435,876 |
|  | CNCM-4574 | NMTX01000009 | Lugh | 13,037-47,111 |
|  |  | NMTX01000025 | Taranis | compl (68,515-122,519) |
|  |  | NMTX01000030 | Mushu4574 | 70,578-107,173 |
| *F. prausnitzii*  Phylogroup II C | CNCM-4542 | NMTS02000001.1 | Lugh4542 | 516,480-552,921 |
|  |  |  | Oengus | 690,827-632,437 |
|  |  |  | Toutatis | compl(774,105-828,829) |
|  | CNCM-4544 | NMTU01000035 | Lugh4544 | 101,198-137,181 |
|  | CNCM-4541 | NMTR00000000 | - |  |
|  | CNCM-4540 | NMTQ01000037 | Lugh4540 | 23-33,008 |
|  | KLE1255 | AECU01000248 | LughKLE1255 | 1-35,806 |
|  |  | AECU01000219/97/98 | ToutatisKLE1255 | concat of 3 contigs |
| Other *F. prausnitzii* | CNCM-4575 | NMTY01000015 | Epona | compl (16,636-66,636) |
|  | L2-6 | FP929045.1 | TaranisL2-6 | 30,658-74,593 |
|  |  |  | LughL2-6 | 633,929-665,157 |
|  |  |  | ToutatisL2-6 | 1,623,846-1,677,432 |
| *Blautia hansenii* | DSM 20583 | NZ_GG698588 | Lagaffe | 179,938-228,023 |
| Unclassified *clostridium* sp. | M62/1 | NZ_GG730315 | Lagaffe | 16,101-65,583 |
| Unclassified *ruminococcaceae* bacterium | D16 | NZ_KI391947 | MushuRBD16 | 124,001-160,259 |
| **Viral contigs and phage or prophage genomes** | | |  | |
| Clone 2204 scaffold 812 | | JQ680370.1 |  |  |
| Clone 2209 scaffold 1451 | | JQ680376.1 |  |  |
| Clone 2200 scaffold 2278 | | JQ680365.1 |  |  |
| Clone 2204 scaffold14 | | JQ680368.1 |  |  |
| FP_Mushu | | MG711460 |  | |
| FP_Lagaffe | | MG711461 |  | |
| FP_Lugh | | MG711464 |  | |
| FP_Taranis | | MG711467 |  | |
| FP_Epona | | MG711462 |  | |
| FP_Toutatis | | MG711466 |  | |
| FP_Brigit | | MG711465 |  | |
| FP_Oengus | | MG711463 |  | |

* : positions with * indicate that they were confirmed by sequencing of encapsidated DNA
