# Supplementary material for: Phages infecting Faecalibacterium prausnitzii belong to novel viral genera that help to decipher intestinal viromes
Source: Microbiome. 2018 Apr 3;6:65. doi: 10.1186/s40168-018-0452-1 (PMC5883640; doi:10.1186/s40168-018-0452-1)
Supplement: Supplementary file 2 — Similarity matrices of phage proteins within each proposed genus. For each pair of phages, the percentage of shared proteins as well as their mean amino acid identity is indicated. For each proposed genus, the closest known phage was included in the analysis, and its host is indicated. Numbers in brackets indicate the number of ORF per genome, and most of the variability within a group is due to annotation errors. Numbers in bold indicate highly homologous prophages, which were considered to correspond to the same species. (DOCX 28 kb) [file 40168_2018_452_MOESM2_ESM.docx]

| **‘FPmushuvirus’** | | Mushu in A2-165 (56) | Mushu in 4543 | Mushu in 4574 | MushuRBD16 |
| --- | --- | --- | --- | --- | --- |
| Mushu in 4543 | Shared proteins | **98 %** |  |  |  |
| (52) | Mean identity | **97,8 %** |  |  |  |
| Mushu in 4574 | Shared proteins | **96 %** | **98 %** |  |  |
| (56) | Mean identity | **97,1 %** | **99,9 %** |  |  |
| MushuRBD16 | Shared proteins | 88 % | 90 % | 90 % |  |
| (48) | Mean identity | 68,6 % | 68,8 % | 68,6 % |  |
| Mu (55) | Shared proteins | 20% | 23% | 21% | 27% |
| *Escherichia coli* | Mean identity | 29,1 % | 29,8 % | 29,8 % | 29,3 % |

| **‘FPlughvirus’** | | Lugh in 4574 | Lugh in 4543 | Lugh4542 | Lugh4544 | LughKLE-1255 | Lugh4540 | LughL2/6 |
| --- | --- | --- | --- | --- | --- | --- | --- | --- |
| Lugh in 4543 (42) | Shared proteins  Mean identity | **100%** |  |  |  |  |  |  |
|  |  | **99.6%** |  |  |  |  |  |  |
| Lugh4542 (43) | Shared proteins | 58% | 57% |  |  |  |  |  |
|  | Mean identity | 60% | 60.6% |  |  |  |  |  |
| Lugh4544 (56) | Shared proteins | 47% | 45% | 0.91 |  |  |  |  |
|  | Mean identity | 61.7% | 62.4% | 91.4% |  |  |  |  |
| LughKLE12 (54) | Shared proteins | 49% | 48% | 73% | 72% |  |  |  |
|  | Mean identity | 57% | 67.5% | 83% | 84.6% |  |  |  |
| Lugh4540 (62) | Shared proteins | 64% | 62% | 52% | 45% | 50% |  |  |
|  | Mean identity | 69.8% | 70.5% | 80.7% | 78.5% | 75,5% |  |  |
| LughL2/6 (35) | Shared proteins | 46% | 46% | 63% | 43% | 43% | 40% |  |
|  | Mean identity | 58.4% | 58.4% | 61% | 51.1% | 53,6% | 53,3% |  |
| BCJA1c (58)  *Bacillus clarkii* | Shared proteins | 35% | 31% | 11% | 11% | 12% | 33% | 20% |
|  | Mean identity | 38,7% | 40.1% | 30,7% | 31% | 30,6% | 41,2% | 28,3% |

| **‘FPtoutatisvirus’** |  | Toutatis  /4542pp3 (89) | L2/6pp3 | KLEpp2 | SL3/3pp1 |
| --- | --- | --- | --- | --- | --- |
| ToutatisL2-6 (72) | Shared proteins | 88% |  |  |  |
|  | Mean identity | 93,34 % |  |  |  |
| ToutatisKLE1255 (89) | Shared proteins | 79% | 78% |  |  |
|  | Mean identity | 92.2 % | 89.3 % |  |  |
| ToutatisSL3/3 (67) | Shared proteins | 55% | 45% | 57% |  |
|  | Mean identity | 52.2 % | 48.5 % | 50.9 % |  |
| Lily (74) | Shared proteins | 16% | 13% | 16% | 16% |
| *Paenibacillus larvae* | Mean identity | 34,3% | 32,6% | 34,2% | 35,6% |

| ‘**FPtaranisvirus’** |  | Taranis in 4574 (89) | Taranis4543 | TaranisL26 |
| --- | --- | --- | --- | --- |
| Taranis in 4543 (65) | Shared proteins | **98%** |  |  |
|  | Mean identity | **99,2 %** |  |  |
| TaranisL2-6 (59) | Shared proteins | 80% | 73% |  |
|  | Mean identity | 86,05 % | 88,1 % |  |
| Lily (74) | Shared proteins | 23% | 23% | 15% |
| *Paenibacillus larvae* | Mean identity | 34,6% | 35% | 34,6% |

| ‘**FPeponavirus’** | |  | Epona in 4573 (76) | Epona in 4575 | EponaM21-2 |
| --- | --- | --- | --- | --- | --- |
| Epona in 4575 (75) | Shared proteins | | **100%** |  |  |
|  | Mean identity | | **99,9 %** |  |  |
| EponaM21-2 (75) | Shared proteins | | 57% | 55% |  |
|  | Mean identity | | 45.2% | 45.5% |  |
| \| X29 \| \| --- \| \| *Vibrio cholerae* \| | Shared proteins | | 30% | 31% | 17% |
|  | Mean identity | | 32% | 32.1% | 33.5% |
