# Supplementary material for: Phages infecting Faecalibacterium prausnitzii belong to novel viral genera that help to decipher intestinal viromes
Source: Microbiome. 2018 Apr 3;6:65. doi: 10.1186/s40168-018-0452-1 (PMC5883640; doi:10.1186/s40168-018-0452-1)
Supplement: Supplementary file 3 — Alignment of Mushu and Lagaffe with close relatives. A) Lagaffe prophages. Remarkably, Lagaffe prophage is present in the genome of B. hansenii DSM20583, only distantly related to F. prausnitzii. Alignment with a viral contig obtained from metagenomic reads suggests that the packaging site is just before the terminase gene. B) Mushu prophage is present in three F. prausnitzii isolates. Its synteny is perfectly conserved with a distantly related prophage in the genome of the Ruminococcaceae bacterium D16 (RBD16). (PPTX 466 kb) [file 40168_2018_452_MOESM3_ESM.pptx]

## Slide 1
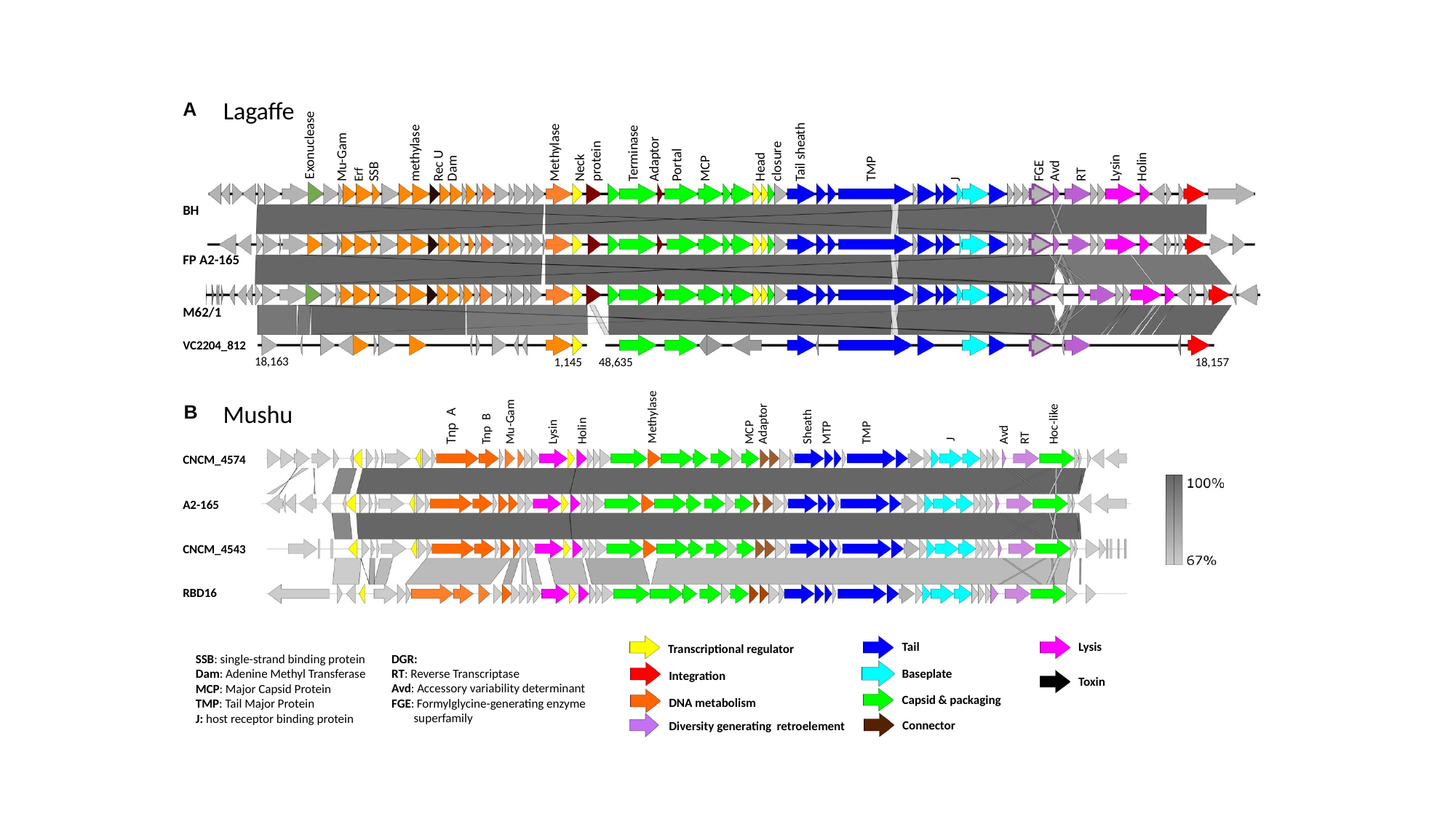

Lagaffe
A
Neck protein
Head closure
Exonuclease
Mu-Gam
methylase
Tail sheath
Methylase
Terminase
FGE
RT
Adaptor
MCP
Dam
SSB
TMP
Lysin
Holin
Erf
Rec U
Portal
Avd
J
BH
FP A2-165
M62/1
VC2204_812
18,163
1,145
48,635
18,157
Mushu
B
Sheath
Lysin
MTP
 Methylase
RT
Mu-Gam
MCP
TMP
Hoc-like
Tnp A
Adaptor
Avd
Tnp B
Holin
 J
CNCM_4574
A2-165
A2-165
CNCM_4543
RBD16
Tail
Lysis
Transcriptional regulator
Baseplate
Integration
Toxin
Capsid & packaging
DNA metabolism
Connector
Diversity generating retroelement
DGR:
RT: Reverse Transcriptase
Avd: Accessory variability determinant
FGE: Formylglycine-generating enzyme
superfamily
SSB: single-strand binding protein
Dam: Adenine Methyl Transferase
MCP: Major Capsid Protein
TMP: Tail Major Protein
J: host receptor binding protein
