# Supplementary figures and images for: Phages infecting Faecalibacterium prausnitzii belong to novel viral genera that help to decipher intestinal viromes
Source: Microbiome. 2018 Apr 3;6:65. doi: 10.1186/s40168-018-0452-1 (PMC5883640; doi:10.1186/s40168-018-0452-1)

## Slide 1
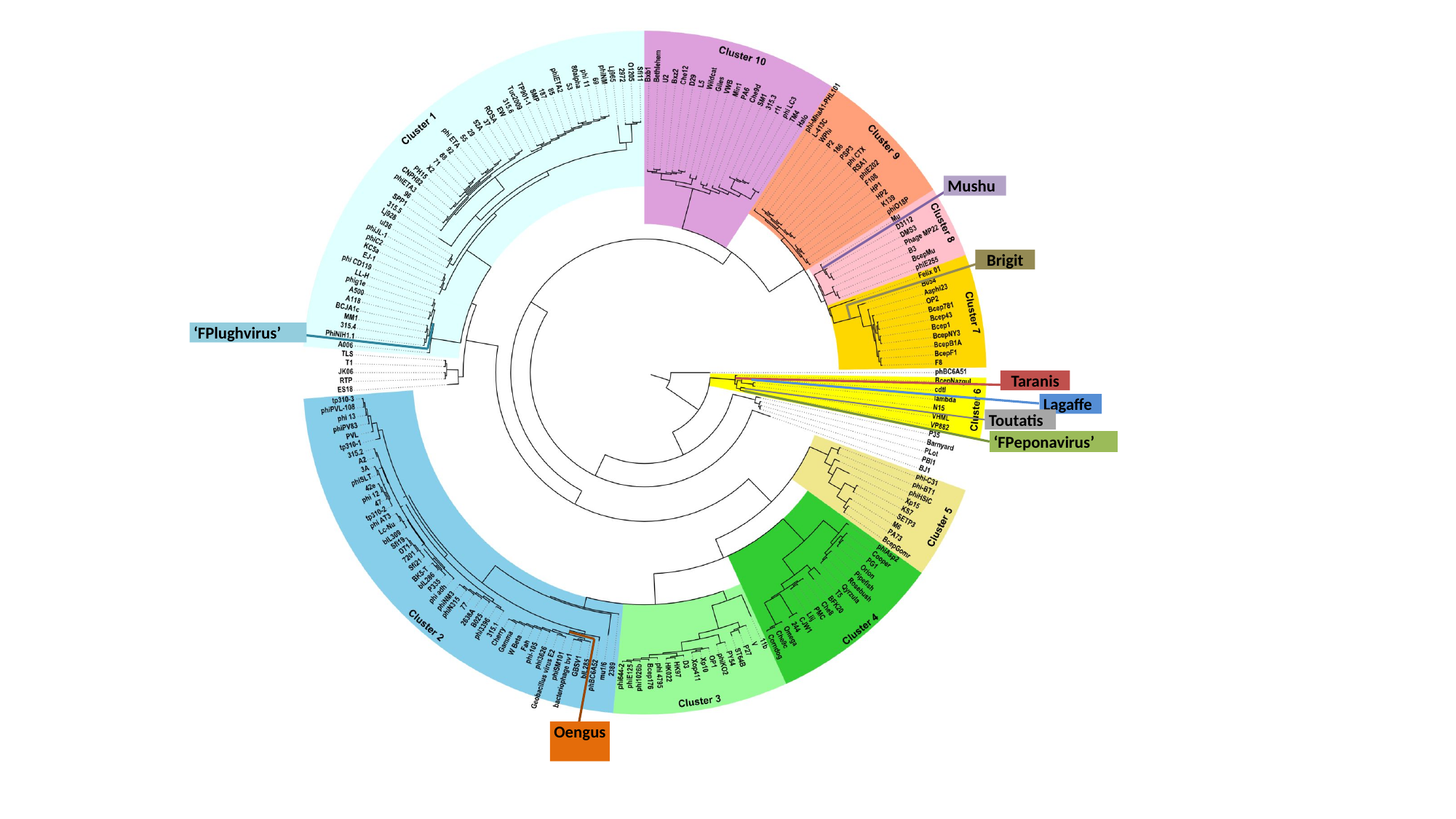

Mushu
Brigit
‘FPlughvirus’
Taranis
Lagaffe
Toutatis
‘FPeponavirus’
Oengus

Supplement: Supplementary file 4 — Classification of F. prausnitzii phage genera by Virfam. Classification of F. prausnitzii phage genera by Virfam (biodev.cea.fr/virfam/). Four genera fall into the less resolved cluster, the cluster 6. (PPTX 913 kb) [file 40168_2018_452_MOESM4_ESM.pptx]

## Slide 1
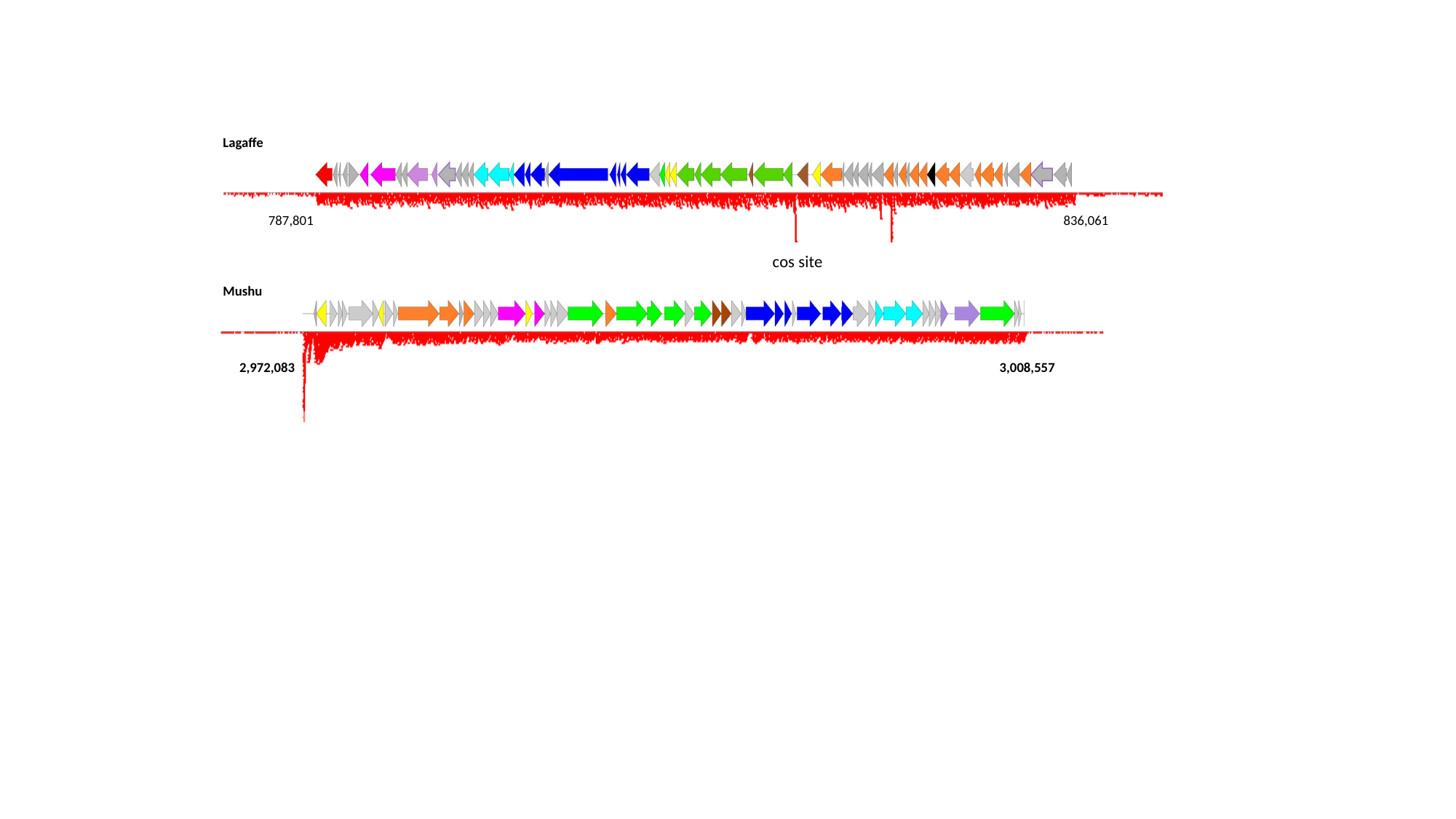

Lagaffe
787,801
836,061
cos site
Mushu
3,008,557
2,972,083

Supplement: Supplementary file 6 — Read coverage of Lagaffe and Mushu prophages. Coverage of the bacterial DNA is represented by surrounding regions of the two prophages. Numbers correspond to positions in the A2-165 genome. Coverage pictures were obtained using Tablet [54]. (PPTX 141 kb) [file 40168_2018_452_MOESM6_ESM.pptx]

## Slide 1
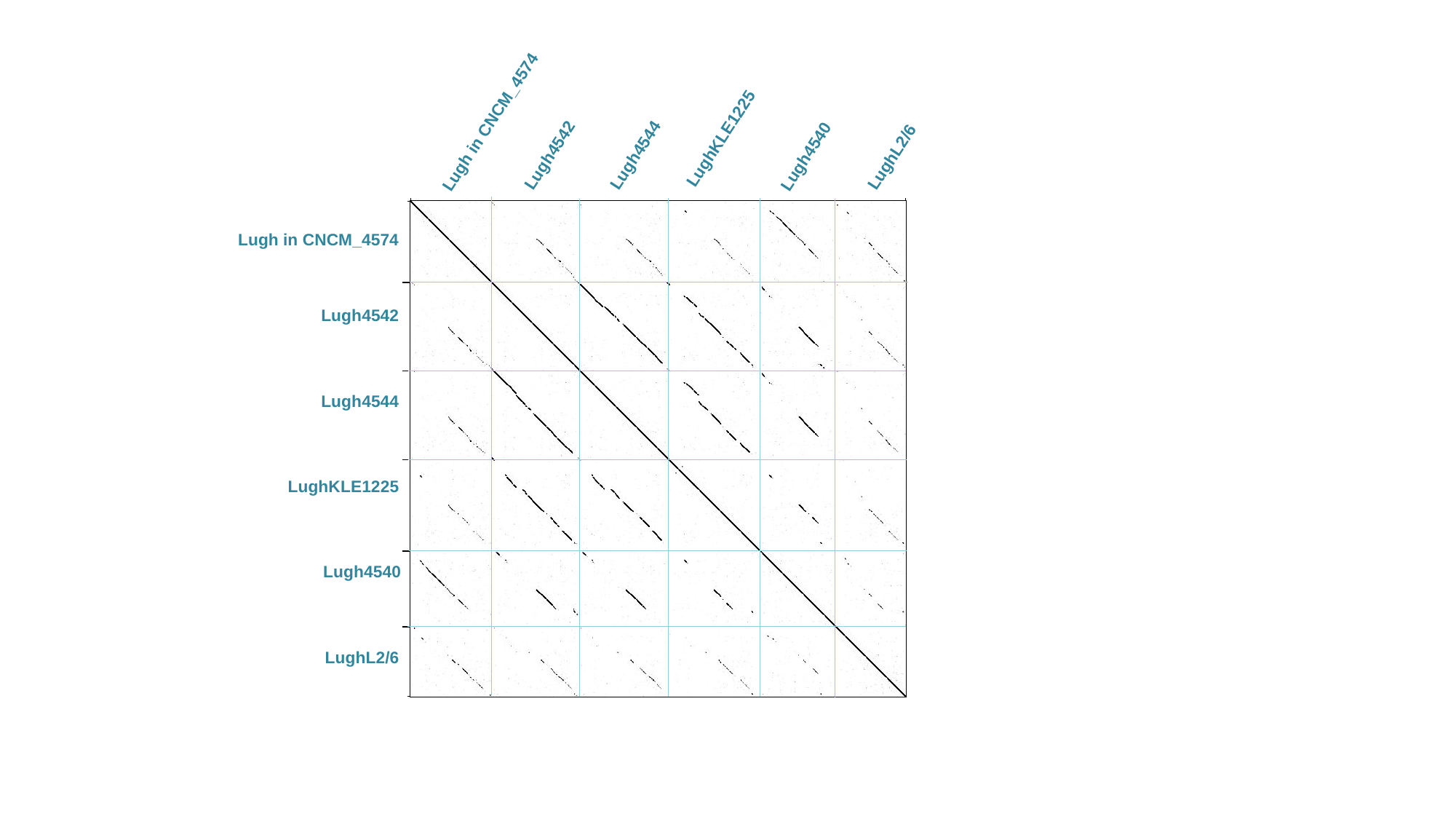

Lugh in CNCM_4574
LughKLE1225
Lugh4542
Lugh4544
LughL2/6
Lugh4540
Lugh in CNCM_4574
Lugh4542
Lugh4544
LughKLE1225
Lugh4540
LughL2/6

Supplement: Supplementary file 9 — Whole genome dotplot of “FPlughvirus” phages. Whole genome dotplot of the six genomes of the proposed “FPlughvirus” genus reveals mosaicism. Phages share regions of high identity interspaced by regions without homology. The dotplot was realized with Gepard. (PPTX 123 kb) [file 40168_2018_452_MOESM9_ESM.pptx]
