# Supplementary material for: Phages infecting Faecalibacterium prausnitzii belong to novel viral genera that help to decipher intestinal viromes
Source: Microbiome. 2018 Apr 3;6:65. doi: 10.1186/s40168-018-0452-1 (PMC5883640; doi:10.1186/s40168-018-0452-1)
Supplement: Supplementary file 5 — DGRs in prophages. A) The position of TR and VR regions in phage genomes and the percentage of nucleotide identity between the sequences (% id), as well as the repeat length, are indicated for each type phage. In all cases, the differences are restricted to adenine residues, and the target genes containing the VR are typical of DGR. B) Genetic organization of DGRs of phages Lagaffe and Mushu, and alignment of their template repeat (TR) and variable region (VR). Red bases highlight point mutation between the two regions. Positions are given relative to the beginning of the prophage, except for Lagaffe (beginning of the encapsidated form of the genome). As in other VR/TR repeats, mismatches concern only adenine residues. C) Mapping of sequence reads of encapsidated phage DNA reveals the high variability in the VR region of both phages. Illustrations were obtained using Tablet [54]. Note that in the case of Lagaffe, the sequence corresponds to the complement of the VR sequence in B). (PPTX 194 kb) [file 40168_2018_452_MOESM5_ESM.pptx]

## Slide 1
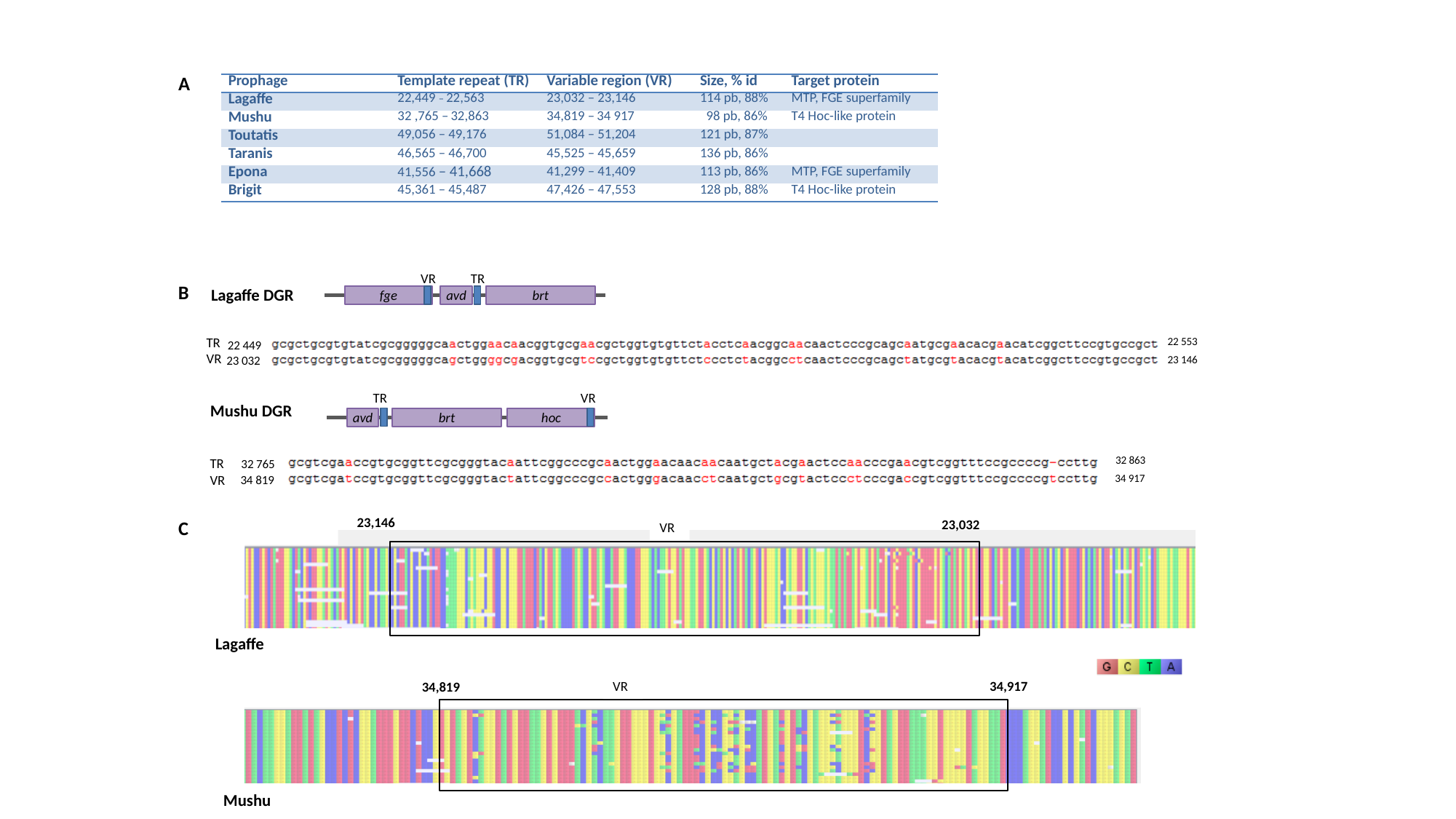

A
| Prophage | Template repeat (TR) | Variable region (VR) | Size, % id | Target protein |
| --- | --- | --- | --- | --- |
| Lagaffe | 22,449 – 22,563 | 23,032 – 23,146 | 114 pb, 88% | MTP, FGE superfamily |
| Mushu | 32 ,765 – 32,863 | 34,819 – 34 917 | 98 pb, 86% | T4 Hoc-like protein |
| Toutatis | 49,056 – 49,176 | 51,084 – 51,204 | 121 pb, 87% | |
| Taranis | 46,565 – 46,700 | 45,525 – 45,659 | 136 pb, 86% | |
| Epona | 41,556 – 41,668 | 41,299 – 41,409 | 113 pb, 86% | MTP, FGE superfamily |
| Brigit | 45,361 – 45,487 | 47,426 – 47,553 | 128 pb, 88% | T4 Hoc-like protein |
VR
TR
B
Lagaffe DGR
fge
avd
brt
TR
VR
40 807
22 553
22 449
23 146
40 224
23 032
TR
VR
Mushu DGR
avd
brt
hoc
32 863
TR
VR
32 765
34 917
34 819
23,146
23,032
C
VR
Lagaffe
34,917
34,819
VR
Mushu
