# Supplementary material for: Phages infecting Faecalibacterium prausnitzii belong to novel viral genera that help to decipher intestinal viromes
Source: Microbiome. 2018 Apr 3;6:65. doi: 10.1186/s40168-018-0452-1 (PMC5883640; doi:10.1186/s40168-018-0452-1)
Supplement: Supplementary file 7 — Lagaffe and Mushu virion concentrations in culture supernatants. Quantification of Mushu and Lagaffe virions in different conditions. Numbers represent means of genomes/ml ± s.e.m. of two to five independent experiments. Absorbance of the culture (O.D600nm) reflects the toxicity of the compound on bacterial growth. (DOCX 13 kb) [file 40168_2018_452_MOESM7_ESM.docx]

|  | Control | Mitomycin C  1 µg/mL | Bile salts 0.1% | Bile salts 0.05% | H_2_O_2_  100 µM | H_2_O_2_  10 µM |
| --- | --- | --- | --- | --- | --- | --- |
| Mushu | 8.0 x 10^6^ ±2.0 x 10^6^ | 8.9 x 10^6^ ±2.3 x 10^6^ | 1.0 x 10^7^ ±9 x 10^6^ | 2.4 x 10^6^ ±9.3 x 10^5^ | 9.4 x 10^6^ ±7.9 x 10^6^ | 4.5 x 10^6^ ±1.5 x 10^6^ |
| Lagaffe | 2.9 x 10^5^ ±2.9 x 10^4^ | 3.9 x 10^5^ ±4.6 x 10^4^ | 5.7.10^4^ ±1.1.10^4^ | 1.4 x 10^5^ ±4.2 x 10^4^ | 7.2 x 10^4^ ±2.3 x 10^3^ | 2.2 x 10^5^ ±2.1 x 10^4^ |
| Final O.D. at 600nm | 1.1±0.1 | 1.1 | 0.05 | 0.55 | 0.15 | 1.05 |
