# Supplementary material for: Phages infecting Faecalibacterium prausnitzii belong to novel viral genera that help to decipher intestinal viromes
Source: Microbiome. 2018 Apr 3;6:65. doi: 10.1186/s40168-018-0452-1 (PMC5883640; doi:10.1186/s40168-018-0452-1)
Supplement: Supplementary file 8 — Lagaffe and Mushu virion concentration in the feces of mice. A) F. prausnitzii concentration (dark blue and dark red) and Mushu virion concentration (light blue and light red) in the feces of mice, as determined by quantitative PCR. DSS treatment does not modify phage and bacteria population levels compared to untreated mice. B) Variation of mice weight with time. Dots represent the average of five mice; bars represent S.E.M. Mice given DSS in drinking water loosed weight, a sign of inflammation. C) Concentration of lipocalin, a marker of intestinal inflammation [55], in mice feces. Concentration was estimated on feces after 7 days of DSS ingestion, using R&D ELISA kit (DY1857) and following manufacturer protocol. The DNA of fecal bacteria was extracted from feces resuspended in 500 μl of PBS. Suspension was centrifuged 1 min at 500g. Supernatant containing bacteria was recovered and mixed with 250 μl of lysis buffer (200 mM NaCl, 20 mM EDTA, 5% SDS), 250 μl of phenol/choloform/isoamyl alcohol (25:24:1;[pH 8.0], Sigma-Aldrich) and half of a tube of silica beads (100 μM MP Bio, Lysing matrix B). Bacteria were lysed using Fast-prep MPBio (5.5, 4 times 30 s). Samples were then centrifuged (13,000g, 3 min, 20 °C) and the aqueous phase was recovered. Four hundred microliters of chloroform/isoamyl alcohol(24:1) was added, mixed vigorously and centrifuged (13,000g, 3 min, 20 °C). DNA was precipitated with two volumes of ethanol and sodium acetate at 0.3 M final. (PPTX 42 kb) [file 40168_2018_452_MOESM8_ESM.pptx]

## Slide 1
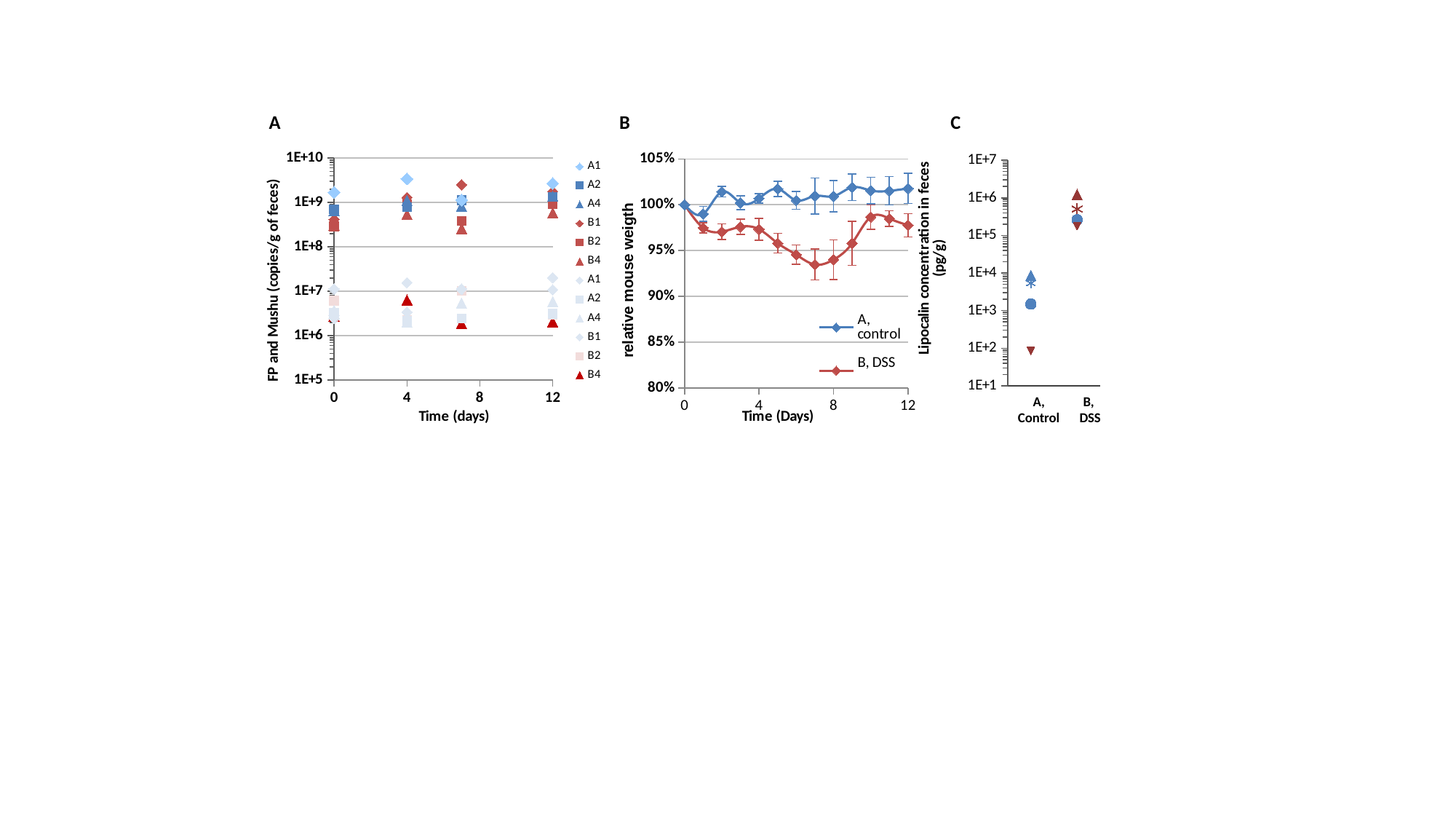

A
B
C
### Chart
| Category | A1 | A2 | A4 | B1 | B2 | B4 | A1 | A2 | A4 | B1 | B2 | B4 |
|---|---|---|---|---|---|---|---|---|---|---|---|---|
### Chart
| Category | A, control | B, DSS |
|---|---|---|
### Chart
| Category | | | | | |
|---|---|---|---|---|---|A,
Control
B,
DSS
