# Supplementary material for: Phages infecting Faecalibacterium prausnitzii belong to novel viral genera that help to decipher intestinal viromes
Source: Microbiome. 2018 Apr 3;6:65. doi: 10.1186/s40168-018-0452-1 (PMC5883640; doi:10.1186/s40168-018-0452-1)
Supplement: Supplementary file 12 — Sequences of oligonucleotides used for quantitative PCR. (DOCX 15 kb) [file 40168_2018_452_MOESM12_ESM.docx]

| **Primer** | **Sequence** (5’-3’) |
| --- | --- |
| FP qPCR F | CGGATCCCAACTGGGTCTAC |
| FP qPCR R | CGGCAGGCGTTTTTGATCTT |
| Lagaffe qPCR F | CTGCCGAAACTATGCGAACG |
| Lagaffe qPCR R | GCGGCGTTTCTTGGACATTT |
| Mushu qPCR F | AAGTTCCTCATGGGACAGCG |
| Mushu qPCR R | TTGCCCTTCATCATCCGCTT |
